# Risk evaluation of cognitive impairment in patients with heart failure: A call for action

**DOI:** 10.1016/j.ijcha.2022.101133

**Published:** 2022-10-10

**Authors:** Sanne Kuipers, Jacoba P. Greving, Hans-Peter Brunner-La Rocca, Rebecca F. Gottesman, Robert J. van Oostenbrugge, Nicole L. Williams, Geert Jan Biessels, L. Jaap Kappelle, Heart-Brain Connection consortium

**Affiliations:** a Department of Neurology, UMC Utrecht Brain Center, University Medical Center Utrecht, Utrecht, The Netherlands; b Julius Center for Health Sciences and Primary Care, University Medical Center Utrecht, Utrecht University, Utrecht, The Netherlands; c Department of Cardiology, Maastricht University Medical Center, Maastricht, The Netherlands; d School of Cardiovascular Diseases CARIM, University Maastricht, Maastricht, The Netherlands; e Stroke Branch, National Institute of Neurological Disorders and Stroke, Intramural Research Program, NIH, Bethesda, MD, USA^1^; f Department of Neurology, Maastricht University Medical Center, Maastricht, The Netherlands; g Department of Neurology, The Johns Hopkins University School of Medicine, Baltimore, MD, USA

**Keywords:** Heart failure, Cognitive impairment, Risk model

## Abstract

**Background:**

Cognitive impairment (CI) is common in patients with heart failure (HF) and impacts treatment adherence and other aspects of patient life in HF. Recognition of CI in patients with HF is therefore important. We aimed to develop a risk model with easily available patient characteristics, to identify patients with HF who are at high risk to be cognitively impaired and in need for further cognitive investigation.

**Methods & results:**

The risk model was developed in 611 patients ≥ 60 years with HF from the TIME-CHF trial. Fifty-six (9 %) patients had CI (defined as Hodkinson Abbreviated Mental Test ≤ 7). We assessed the association between potential predictors and CI with least-absolute-shrinkage-and-selection-operator (LASSO) regression analysis. The selected predictors were: older age, female sex, NYHA class III or IV, Charlson comorbidity index ≥ 6, anemia, heart rate ≥ 70 bpm and systolic blood pressure ≥ 145 mmHg. A model that combined these variables had a c-statistic of 0.70 (0.63–0.78). The model was validated in 155 patients ≥ 60 years with HF from the ECHO study. In the validation cohort 51 (33 %) patients had CI (defined as a Mini Mental State Exam ≤ 24). External validation showed an AUC of 0.56 (0.46–0.66).

**Conclusions:**

This risk model with easily available patient characteristics has poor predictive performance in external validation, which may be due to case-mix variation. These findings underscore the need for active screening and standardized assessment for CI in patients with HF.

## 1 Introduction

Cognitive impairment (CI) is increasingly recognized as a common complication and comorbidity in patients with heart failure (HF), with reported prevalence rates varying from 10 to 79 % [1], [2]. CI encompasses not only difficulties in memory, but also difficulties in problem solving and decision-making, attention and production, and comprehension of language. The severity of CI can range from mild impairment to dementia. The exact pathogenesis is unclear, but shared vascular risk factors [3] and both haemodynamic and thrombo-embolic complications of HF have been suggested as potential causes [4]. The number of patients with HF and CI will likely increase due to the aging of the population.

CI may lead to significant difficulties in patients with HF, including interference with self-care requirements for optimal HF management (e.g., symptom monitoring, dietary compliance and medication adherence) [5]. Moreover, CI carries an increased risk of hospitalization and mortality in patients with HF [6], [7], [8]. Yet, CI is often missed in these patients, which may be caused by underestimation of the deficits by physicians [9], [10] and the fact that many patients do not express cognitive complaints themselves [11]. Although clinical guidelines in HF have begun to mention the importance of CI [12], [13], this is not widely adopted in clinical practice. Moreover, guidance regarding when and in which patients cognitive testing should be performed is lacking.

Recognition of patients with HF who are at high risk for CI could help to improve management in these patients, including optimal pharmacotherapy, correction of vascular risk factors, use of medication adherence aids, tailored self-care advice and involvement of family and care-givers in the patient care [12], [14]. Future randomized controlled trials should validate whether pharmacological treatments (e.g. sacubitril [15] or empagliflozin [16]) and non-pharmacological treatments (e.g. physical activity [2]) are beneficial for these patients.

In this study we aimed to develop a risk model that includes easily available patient characteristics, that may aid cardiologists to identify those patients with HF who are at high risk for CI and in need for further cognitive investigation.

## 2 Methods

We developed a risk model in 611 patients with HF from the TIME-CHF (Trial of Intensified versus standard Medical therapy in Elderly patients with Congestive Heart Failure), a prospective randomized controlled trial including patients aged 60 years or older with a NYHA class II or higher, who had elevated *N*-terminal BNP levels and had been hospitalized for HF within the last year [17]. CI was defined as Hodkinson Abbreviated Mental Test (AMT) ≤ 7 [18]. Candidate predictors for the presence of CI (including sociodemographic characteristics, HF related characteristics, psychosocial factors, co-morbidities and vital signs at physical examination) were preselected based on the literature. We screened 13 papers [9], [19], [20], [21], [22], [23], [24], [25], [26], [27], [28], [29], [30] and selected variables that were found to be associated with CI with a p-value < 0.20 in multivariable analyses. Candidate predictors that were available in the TIME-CHF study were: older age, female sex, NYHA class III or IV, coronary artery disease as main cause of HF, preserved left ventricular ejection fraction, higher Charlson comorbidity index (pre-dominantly the presence of cerebrovascular disease, peripheral vascular disease, pulmonary disease and renal failure), depression, anemia, higher heart rate and higher systolic blood pressure. Restricted cubic spline functions and graphs were used to determine whether continuous variables could be analyzed as linear terms or needed transformation. With least-absolute-shrinkage-and-selection-operator (LASSO) regression analysis, we assessed the relation between potential predictors and CI, performed beta shrinkage and predictor selection. The model was externally validated in 155 patients with HF, aged 60 years or older from the ECHO (Evaluating Cognition in Heart Failure within an Outpatient setting) study. In the validation cohort, CI was defined as defined as a Mini Mental State Exam (MMSE) ≤ 24 [31]. Performance was assessed with c-statistics and calibration plots.

## 3 Results

Table 1 shows the clinical characteristics of both the development and external validation cohort. In the development cohort 9 % patients had CI whereas this percentage was 33 % in the external validation cohort. Patients from the development cohort were older and more often had a reduced left ventricular ejection fraction (LVEF) than the external validation cohort. In contrast, patients from the external validation cohort had more co-morbidities such as a higher BMI and more often a Charlson comorbidity index ≥ 6, anemia, atrial fibrillation, diabetes, COPD, history of stroke and depression.

**Table 1:** Clinical characteristics in the development cohort and external validation cohort.

	**Development cohort**			**External validation cohort**		
	**Overall cohort** **(n = 611)**	**CI** **(n = 56)**	**No CI** **(n = 555)**	**Overall cohort** **(n = 155)**	**CI** **(n = 51)**	**No CI** **(n = 104)**
**Demographics**						
Age, mean (SD)	76.9 (7.6)	79.0 (7.4)	76.7 (7.6)	72.5 (8.7)	75.0 (9.1)	71.3 (8.5)
Female sex, n (%)	246 (40 %)	35 (63 %)	211 (38 %)	74 (48 %)	25 (49 %)	49 (47 %)
BMI (kg/m2), mean (SD)	25.6 (4.4)	24.7 (4.8)	25.7 (4.4)	35.6 (8.9)	34.2 (7.6)	36.3 (9.4)
**Heart failure characteristics**						
NYHA class III/IV, n (%)	464 (76 %)	48 (86 %)	416 (75 %)	93 (60 %)	31 (61 %)	62 (60 %)
LVEF (%), mean (SD)	34.9 (13.0)	37.6 (15.3)	34.7 (12.7)	50.3 (16.3)	53.3 (16.2)	48.8 (16.2)
LVEF ≥ 45 %, n (%)	120 (20 %)	16 (29 %)	104 (19 %)	106 (68 %)	37 (73 %)	69 (67 %)
Main cause of heart failure						
Coronary artery disease, n (%)	324 (53 %)	27 (48 %)	297 (54 %)			
Dilated cardiomyopathy, n (%)	88 (14 %)	9 (16 %)	79 (14 %)			
Valvular heart disease, n (%)	23 (4 %)	2 (4 %)	21 (4 %)	n/a	n/a	n/a
Hypertensive heart disease, n (%)	169 (28 %)	17 (30 %)	152 (27 %)			
Other, n (%)	7 (1 %)	1 (2 %)	6 (1 %)			
**Co-morbidities**						
Charlson co-morbidity index ≥ 6, n (%)*	57 (9 %)	10 (18 %)	47 (8 %)	67 (43 %)	25 (49 %)	42 (40 %)
Anemia, n (%)**	171 (28 %)	23 (41 %)	148 (27 %)	103 (66 %)	35 (69 %)	68 (65 %)
Atrial fibrillation, n (%)	204 (34 %)	20 (36 %)	184 (33 %)	85 (55 %)	30 (59 %)	55 (53 %)
Diabetes, n (%)	215 (35 %)	23 (41 %)	192 (35 %)	83 (54 %)	30 (59 %)	53 (51 %)
Renal failure, n (%)	349 (57 %)	32 (57 %)	317 (57 %)	n/a***	n/a	n/a
COPD, n (%)	121 (20 %)	11 (20 %)	110 (20 %)	48 (31 %)	13 (25 %)	35 (34 %)
History of stroke, n (%)	51 (8 %)	7 (13 %)	44 (8 %)	21 (14 %)	10 (20 %)	11 (11 %)
Depression, n (%)	79 (13 %)	8 (14 %)	71 (13 %)	83 (54 %)	27 (53 %)	56 (54 %)
**Physical examination**						
Heart rate ≥ 70 bpm, n (%)	389 (64 %)	42 (75 %)	347 (63 %)	80 (52 %)	31 (61 %)	49 (47 %)
SPB ≥ 145 mmHg, n (%)	71 (12 %)	11 (20 %)	60 (11 %)	24 (15 %)	9 (18 %)	15 (15 %)

Data is expressed as mean (standard deviation) or number (%).

CI, cognitive impairment; LVEF, left ventricular ejection fraction; NYHA New York Heart Association; SPB, systolic blood pressure.

*** Patients with creatinine > 2.5 were excluded in the ECHO study.

* We used the classical Charlson co-morbidity index (Charlson 1987).

** Anemia was defined as a hemoglobin concentration of < 12 g/dl for women and < 13 g/dl for men.

**Fig. 1:**
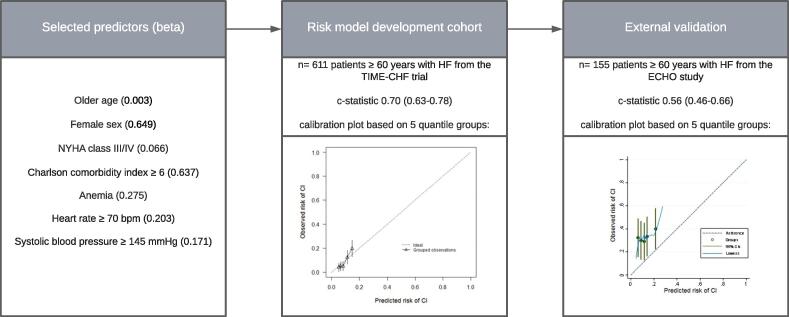
Summary of main findings. CI, cognitive impairment; HF, heart failure. Beta = beta coefficient from LASSO regression analysis.

From the available candidate predictors we selected seven predictors with LASSO regression analysis: older age, female sex, NYHA class III or IV, Charlson comorbidity index ≥ 6, anemia, heart rate ≥ 70 bpm and systolic blood pressure ≥ 145 mmHg. A model that combined these predictors had a moderate to good performance (c-statistic 0.71, 95 % CI 0.63–0.78) with overall observed risks within the range of the expected risks. Unfortunately, external validation revealed poor performance of the model (c-statistic 0.56, 95 % CI 0.46–0.66) and poor calibration. The main findings are summarized in Fig. 1.

## 4 Discussion

These results confirm that patients with HF are vulnerable for CI, with a prevalence of CI varying from 9 % in the development cohort to 33 % in the external validation cohort. The wide range in prevalence of CI has also been described in previous studies [1] and is most likely caused by heterogeneity in both the type and severity of HF and the method of cognitive assessment. HF is a heterogenous syndrome with a large variety in subtypes and co-morbidities. Also, large differences exist in methodology of cognitive assessment in patients with HF [1], [32].

Although both cohorts were comparable in age and severity of HF, we consider the heterogeneity in type of HF and related co-morbidities between our two cohorts, also referred to as ‘case mix differences’, as the main reason why we could not reproduce the results of our original risk model in the external validation cohort. Case-mix variation across cohorts can lead to genuine differences in performance of risk models [33]. Heterogeneity between cohorts of patients with HF is also the most probable reason why previous studies that focused on variables related to CI in patients with HF show conflicting results [34]. A limitation of our study is the lack of more clinical details (e.g. educational level and information about the etiology of HF in the external validation cohort).

A strength of this study is the prospective nature of both the development cohort and external validation cohort. Another strength is the preselection of candidate predictors, that have been previously associated with CI in patients with HF. In this way, we have avoided selecting of predictors purely on statistical significance in the development cohort.

In addition to the aforementioned differences in type of HF and related co-morbidities between the development and external validation cohort, some other limitations need to be addressed. First, the risk model is developed in a trial population. Therefore, our cohort may not be representative of the entire HF population because of application of strict inclusion and exclusion criteria in trial participants. This selection bias may influence the clinical applicability of our findings. Second, although candidate predictors were selected based on previous literature, we may have missed predictors that could improve the performance of the risk model, but were not available in the development cohort (e.g. educational level). Third, although cognitive assessment with the AMT seems to be comparable to the MMSE [35] ideally CI should be diagnosed with the same cognitive assessment scales. Moreover, the Montreal Cognitive Assessment (MOCA) is a more sensitive screenings test for CI in patients with HF than the AMT or MMSE [32]. Last, because the number of outcomes in the development cohort was small, our risk model may be overfitted. Overfitting may lead to underestimation of the probability of CI in low-risk patients and overestimation of the probability of CI in high-risk patients. However, LASSO regression analysis is known to reduce this problem and to develop more accurate risk models [36].

This study underscores that detecting CI is important, but clinically challenging in patients with HF. It emphasizes the need for active screening for CI in individual patients with HF. Development of strategies to identify patients with HF at high risk for CI are required. Such cognitive risk evaluation should be sensitive, but also efficient to enable implementation in clinical practice. For studies to develop cognitive risk models in patients with HF, a standardized assessment of CI is required, as well as a broad range of variables related to HF. Furthermore, bigger datasets are needed to account for differences in the patient cohorts of HF.

## 5 Conclusions

Results of this risk model with easily available patient characteristics, to identify patients with HF who are at high risk to be cognitively impaired, could not be reproduced in an external validation cohort, which may be due to case-mix variation. Our findings underscore the need for active screening and standardized assessment for CI in patients with HF.

## Declaration of Competing Interest

The authors declare that they have no known competing financial interests or personal relationships that could have appeared to influence the work reported in this paper.
